# 24-month clinical outcomes of a treat-and-extend regimen with ranibizumab for wet age-related macular degeneration in a real life setting

**DOI:** 10.1186/s12886-017-0451-1

**Published:** 2017-04-27

**Authors:** Athanasios Vardarinos, Nitin Gupta, Raazia Janjua, Abigail Iron, Theo Empeslidis, Konstantinos T. Tsaousis

**Affiliations:** 1 Eye Treatment Centre, West Suffolk Hospital, Bury St Edmunds, UK; 2 Department of Ophthalmology, Leicester Royal Infirmary, University Hospitals of Leicester, Leicester, UK

**Keywords:** Ranibizumab, Treat and extend, Age related macular degeneration

## Abstract

**Background:**

To evaluate the clinical effectiveness and analyze the outcomes of a treat-and-extend (T&E) treatment regimen with ranibizumab for wet age-related macular degeneration (ARMD) in real life clinical settings over the first 2 years (24 months) of treatment.

**Methods:**

Retrospective analysis of visual acuity, spectral domain optical coherence tomography (SD-OCT) parameters and treatment burden data of 56 eyes of 54 unselected treatment naive patients diagnosed with exudative ARMD. Monthly injections were offered until no signs of disease activity such as intra-retinal (IRF) or sub-retinal fluid (SRF) were evident on SD-OCT, followed by a gradual extension of the treatment interval by 2 weeks until a maximum of 12 weeks.

**Results:**

The study met its main objective, demonstrating a mean best-corrected visual acuity gain of 8.3 letters (mean 68.8 ± 11) at month 12 and 5.2 letters (mean 65.7 ± 12.3) at 24 months compared to baseline (mean 60.5 ± 8.9). Anatomical improvement was also documented with a mean reduction of central retinal thickness by 139.7 μm at 24 months (244.9 ± 48.3) compared to baseline (384.6 ± 154.9). Forty-seven eyes (83.9% *N* = 56) gained vision or preserved baseline vision with 23 eyes (41.1%) gaining 10 letters or more at month 12. Out of the 46 eyes that completed 24 months of treatment and monitoring, 27 (58.7% *N* = 46) kept a BCVA above baseline with 18 of those (39% *N* = 46) maintaining a 10-letter gain throughout the 24 months. Six eyes (13% *N* = 46) lost more than 10 letters by month 24. The mean number of injections was 12.1 ± 2.8 over the 24-month period. Twenty-seven eyes (55.1% *N* = 56) achieved a treatment interval of 10 weeks or more at month 12, while the respective number at month 24 was 20 eyes (43.4% *N* = 46) in addition though to four more patients (8.7% *N* = 46) who were not receiving injections at month 24 since they were placed on a Monitor & Extend regime.

**Conclusions:**

This is the first UK real-life study of a T&E treatment protocol with ranibizumab for exudative ARMD in a 24-month period and suggests that such a regimen is clinically effective and can achieve favourable outcomes with a significant reduction of the treatment burden compared to monthly PRN.

## Background

It is well established that age-related macular degeneration (ARMD) is one of the leading causes of vision loss in the United Kingdom (UK) and worldwide [1].

The advent of the intraocular use of pharmaceutical agents inhibiting the vascular endothelial growth factor (VEGF) in the last 10 years has revolutionized the treatment of the exudative forms of ARMD and has resulted to favourable clinical outcomes compared to previously available treatment options [2]. Randomized clinical trials studying the intravitreal use of ranibizumab [2, 3], aflibercept [4] and the off-label use of bevacizumab [5, 6] reported significant improvement in terms of visual acuity gains and anatomical stability. In the UK, the National Institute for Health and Care Excellence (NICE) published its guidelines for the treatment of exudative ARMD in the National Health Service (NHS) with Ranibizumab in 2008 [7] and with Aflibercept in 2013 [8].

Since the publication of the pivotal ANCHOR and MARINA randomised controlled trials of monthly treatment regimen, researchers have studied the implementation of treatment protocols with fixed dosing [9–11] or as per needed injections with monthly monitoring [12] but less frequent treatment in order to achieve similar clinical results with a decreased treatment burden. The PRONTO study demonstrated that monthly monitoring with as-per-needed treatment (PRN) would offer visual results comparable to monthly injections [12, 13] and has led to the adoption of such protocols in many ophthalmic units. The need for an individualized treatment approach saw the development of treat and extend (T&E) protocols where the decision for each injection and the treatment interval is based on the response of the patient to the last injection [14, 15]. Such treatment approaches are gaining popularity among retina specialists and the published results on vision and disease stability show it is a safe and efficient treatment approach [16, 17].

This retrospective study aims to present clinical outcomes of a ranibizumab T&E protocol offered to a cohort of patients with exudative ARMD in a non-study population over a period of 24 months.

## Methods

This study was conducted in a National Health Service (NHS) Secondary Ophthalmology Department in England (Bury St Edmunds, West Suffolk) and following a decision to change the treatment strategy for newly diagnosed patients with wet ARMD on ranibizumab from PRN into T&E in November 2013. The study is retrospective in nature conducted in October 2016 according to the local clinical governance procedure and protocols. The clinical notes and OCT data of patients diagnosed with wet ARMD who were offered ranibizumab (0.05 mg/0.5 ml) intravitreal injections on a T&E between November 2013 and September 2014 were reviewed. Treatment was initiated when the best corrected visual acuity (BCVA) was between 0.3 and 1.20 Log MAR (70 to 25 EDTRS letters) or 6/12 to 6/96, there was not any permanent structural damage to the fovea and the lesion size was less or equal to 12 disk diameters as per the NICE guidelines for treatment of exudative ARMD in the UK NHS [7]. Baseline EDTRS BCVA testing at 4 m, SD-OCT examination using the Cirrus 5000 system (Zeiss, Germany) and fundus fluorescein angiography were performed and the patients were offered monthly injections, every 28 days until a dry macula was evident on SD-OCT.

Once that was achieved, the injections continued, incrementally increasing the treatment intervals by 2 weeks after each injection until a maximum interval of 12 weeks provided there were no signs of recurrence of disease activity on SD-OCT or BCVA loss of more than 5 letters which could not be explained by other ophthalmic conditions. Injections were offered at every visit, BCVA measured and SD-OCT performed at each visit. In case of a recurrence the treatment interval was reduced by 2 weeks. Signs of disease activity on SD-OCT were considered to be the presence of intra-retinal (IRF) and/or sub-retinal (SRF) fluid and/or retinal/sub-retinal haemorrhage. The presence of a pigmentary epithelial detachment without IRF or SRF did not classify as active exudation. In the second year of treatment, for patients who achieved repeated treatment intervals of 12 weeks ﻿and on the review visit, the condition was considered inactive (no IRF or SRF, no haemorrhage, no BCVA loss compared to last visit) the patients were given the option to be placed on a monitor and extend (M&E) regime, where they would be reviewed with an SD-OCT on incrementally increasing intervals (2–3 weeks at a time) and would be offered an injection only if there was disease recurrence. In cases of bilateral involvement, each eye was treated individually. If appropriate, an injection in both eyes was given at the same visit.

The primary objective of this study was to document the improvement on EDTRS BCVA 12(±1) and 24(±1) months after the initiation of treatment for each patient. Secondary objectives were to document the improvement on EDTRS BCVA at month 3 after three mandatory monthly injections, the number of injections per patient over the 12 month and 24 month period, the mean improvement of the central retinal thickness by means of SD-OCT at months 3, 12 and 24, the percentage of patients gaining or losing more than 10 letters at 12 and 24 months, the percentage of patients achieving a treatment interval of 10 weeks or more at month 12 and 24 and the documentation of injection related complications.

### Statistical analysis

All data were collected retrospectively and analyzed using Microsoft Excel 2013 for Windows (Microsoft Corporation, Redmond, WA, USA) and SPSS version 16.0 for Windows (SPSS Inc., Chicago, IL, USA). Analyses of variance with two-sided paired Student’s *t* tests were performed. The level of statistical significance for the *P* value was set to less than .05.

## Results

A total of 60 treatment naïve patients diagnosed with exudative ARMD between November 2013 and September 2014 and placed on T&E ranibizumab were identified. Four patients were lost to follow-up and did not complete at least 12 months of treatment. A cohort of 56 eyes of 54 patients who completed 12 months and 46 eyes of 45 patients who completed 24 months of treatment on T&E or M&E is included in this study. Nine patients were lost to follow-up or died before the 24 months.

Mean age of the patients at presentation was 80.9 ± 8.8 years (age range 57–96), 36 (66.7%) of them were female and 23 eyes (41%, *N* = 56) were pseudophakic at baseline (Table 1).

**Table 1:** Patient demographics and baseline characteristics

Number of patients	54
Number of eyes	56
Mean age in years at diagnosis (SD, range)	80.9 ± 8.8 (57–96)
Male: Female gender	18: 36
Number of eyes pseudophakic at baseline	23
Right: Left eye	28: 28
Mean EDTRS BCVA at diagnosis (SD, range)	60.5 ± 8.9 (35–70)
Mean CRT at diagnosis in μm (SD, range)	384.6 ± 154.9 (210–1193)

### Clinical outcomes – Visual acuity

**Fig. 1:**
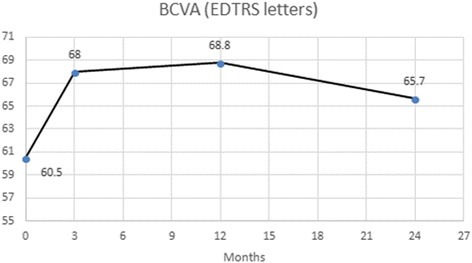
Clinical outcomes on visual acuity over 24 months Note that VA is slightly decreased at 24 months compared to 12-months time point but still significantly better than presentation

The baseline mean ETDRS BCVA was 60.5 ± 8.9 letters ranging from 35 to 70 letters. At month 3 following three mandatory injections for all the patients the mean BCVA had risen to 68 ± 8.7 letters (range 45 to 82 letters) and there was further improvement with the mean BCVA at 68.8 ± 11.1 letters (range 33 to 84 letters) at month 12, with an average gain of 8.3 letters from baseline (*p* < 0.001). At the end of the study period at month 24 the improvement on vision compared to baseline was maintained with a mean BCVA of 65.7 ± 12.3 letters (range 31 to 85), a gain of 5.2 letters (*p* = 0.007). No statistical difference was found between BCVA at the end of year 1 and the end of second year (*p* = 0.18). At month 12, 45 eyes (80.4% *N* = 56) gained vision and three (5.3% *N* = 56) had kept their baseline BCVA, with 23 eyes (41% *N* = 56) gaining 10 letters or more, while at month 24 twenty-seven eyes (58.7% *N* = 46) maintained BCVA above baseline with 18 eyes still gaining more than 10 letters (39.1% *N* = 46). At month 12 eight eyes (14.3% *N* = 56) lost vision compared to baseline with two losing more than 10 letters (3.5% *N* = 56). By month 24, 19 eyes lost vision with nine of those eyes (18.4% *N* = 46) though staying within 5 letters of their baseline vision and only six eyes (13% *N* = 46) losing more than 10 letters. (Fig. 1) Six eyes (10.7% *N* = 56) developed clinically significant age-related cataract and underwent phacoemulsification with an intraocular lens implant before the end of the study period.

### Clinical outcomes – Anatomical improvement

**Fig. 2:**
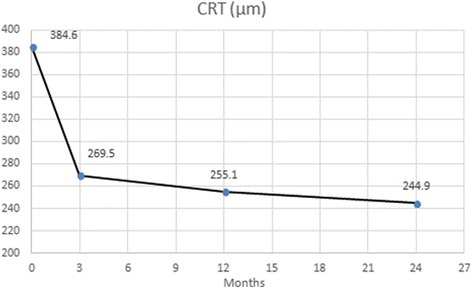
Clinical outcomes on central retinal thickness over 24 months. CRT was even better at 24 months than 12 months

Treatment with T&E ranibizumab for this cohort of eyes, led to the decrease of central retinal thickness from a mean of 384.6 ± 154.9 μm, (range 210–1193 μm, *N* = 56) on SD-OCT at baseline to a mean of 255.1 ± 49.8 μm, (range 176–817 μm, *N* = 56) at month 12 (*p* < 0.001) and to a mean of 244.9 ± 48.3 μm, (range 173–598 μm, *N* = 46) at month 24, an average reduction of −139.7 μm from baseline (*p* < 0.001). (Fig. 2).

### Clinical outcomes – Treatment burden

**Fig. 3:**
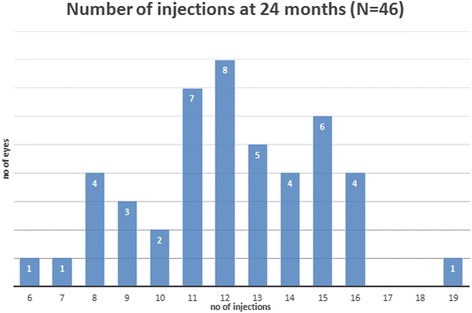
Distribution of the number of injections per eye at month 24 (revised)

**Fig. 4:**
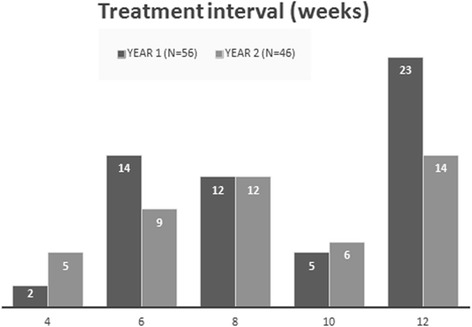
Distribution of the treatment interval per eye at month 12 and 24

The average number of injections per eye over the 12-month period was 7.75 ± 1.3, (range 5 to 11) and 28 eyes (50% *N* = 56) had achieved a treatment interval of 10 weeks or more. At the end of the study period at month 24, the average number of injections per eye was 12.1 ± 2.8 (range 6 to 19) and 20 eyes (43.5% *N* = 46) were receiving injections on an interval of 10 weeks or more in addition to four more eyes (8.7% *N* = 46) for whom treatment was discontinued after reaching the 12-week interval and they were placed on a monitor and extend regimen **(**Figs. 3 and 4 **).**

### Clinical outcomes – Adverse events

There were no cases of serious ocular (endophthalmitis, intraocular inflammation, iatrogenic cataract, vitreous haemorrhage, retinal detachment, raised intraocular pressure) or systemic adverse events documented for this cohort of patients.

## Discussion

The T&E treatment strategies are gaining popularity worldwide and it is now the preferred treatment approach for exudative ARMD for the majority of retina specialists [17]. This has been facilitated by the need to provide the patients with an individualized, safe and clinical effective treatment regime which at the same time would be cost-effective and would allow the healthcare providers to cope with the increasing demand of ophthalmic services [15]. Recently, there has been a gradual swift from the mainstay approach of monthly monitoring and as-per-needed treatment to T&E protocols [15–18].

In this retrospective trial, it was demonstrated that there was a significant reduction in the number of hospital appointments necessary with an average of 12.1 injections per patient over the 24-month period as opposed to 24 visits/injections per patient with a monthly monitoring and as-per-needed treatment protocol, with an average gain of 8.3 and 5.2 EDTRS letters at months 12 and 24 respectively.

In the largest prospective T&E trial to date by Berg et al., 441 patients were randomized to receive ranibizumab or bevacizumab on a T&E protocol with an incremental two-weekly increase of the treatment interval to a maximum of 12 weeks. They reported a gain of 8.2 EDTRS letters at 12 months and 6.6 EDTRS letters at month 24 for the patients on the ranibizumab arm, a result achieved with an average of 8.0 injections per patient in the first year and a total of 16.0 injections by the end of year 2 [19, 20].

Abedi et al., using a similar T&E protocol reported 2 year results with visual gains on month 12 and 24 analogous to those documented at the pivotal monthly treatment ANCHOR and MARINA trials [16]. Also, Arnold et al., in a retrospective analysis of data supplied by the Fight Retinal Blindness observational registry, demonstrated favourable outcomes with T&E regimen when the introduction of SD-OCT in clinical practise allowed earlier detection of recurrences and the application of strict retreatment criteria [18]. A recently published prospective randomized controlled clinical trial by Wickoff et al., underlines the effectiveness of T&E protocols over monthly management, with the patients on the T&E arm achieving similar clinical outcomes with those on monthly treatment but with fewer injections [17]. The positive effect of T&E regimen in achieving and maintaining visual and anatomic improvements can be present up to 3 years of treatment, as was reported by Rayess N et al. [21].

Notwithstanding the very encouraging and favourable clinical results of this trial for the majority of the patients, there were cases where vision was lost despite achieving a “dry” macula on SD-OCT. This is a known fact from previous studies and it has been suggested that this outcome could be the result of progressive macular atrophy [17, 22, 23]. In our study there were six patients that lost more than 10 letters. Sub-foveal fibrosis and atrophy was the main reason for this outcome, but for one patient (−16 letters) the vision loss was potentially reversible since the patient did develop significant age-related cataract during the course of the study period. The above causes for significant vision loss in patients who initially respond to anti-VEGF treatment have been identified elsewhere in the literature [24], although a recent study did not identify any significant adverse influence of treatment with Ranibizumab in the progression of macular atrophy [25]. Also, there were some patients who lost a few letters of vision despite receiving injections as per T&E and without evidence of progressive atrophy on SD-OCT. Shorter intervals between injections in the second year of treatment which would result in a timelier respond to recurrences and an inevitably increased number of injections would have potentially improved outcomes [20].

As with similar studies [15], the retrospective nature of this single-site study and the limited number of patients are the main limitations. Additionally, it was conducted on a non-study population where the strict adherence to fixed reviews and end-points is not always possible. As a result of the above limitations, direct comparison with larger randomised clinical controlled studies is rather difficult.

## Conclusion

This study demonstrates that a safe and effective alternative to monthly monitoring is feasible in a non-study population and the reduction of treatment burden while maintaining favourable clinical outcomes is achievable through a T&E protocol. This is in agreement with recently published data on long-term outcomes on wet ARMD where the authors suggested a potential superiority of T&E protocols over PRN dosing [24]. Similarly switching from PRN to a T&E approach seems to improve outcomes in patients already receiving treatment [26, 27]. Overall, less monitoring and favourable clinical results can have an advantageous effect for patients and their caregivers and reduce the healthcare resource burden [26, 28]. A recently published review of real-life studies where Ranibizumab was offered as treatment for wet ARMD on PRN of fixed dosing regimens confirmed a constant report of worse outcomes compared to the randomised clinical trials and a suggestion of possible better results in real-life with a T&E approach was made [29]. To the authors best knowledge this is the first study in the UK to report 24-month data on T&E in exudative ARMD. Certainly, more studies are required to report retrospective analyses on the beneficial impact of this treatment strategy change in real-life settings.

## Abbreviations

T&E: Treat and extend
ARMD: Age-related macular degeneration
SD-OCT: Spectral-domain optical coherence tomography
BCVA: Best-corrected visual acuity
